# Factors associated with delayed bleeding following ampullectomy: A retrospective cohort study

**DOI:** 10.1002/deo2.70078

**Published:** 2025-02-14

**Authors:** Katarzyna M. Pawlak, Kareem Khalaf, Sunil Gupta, Daniel Tham, Joseph Chon, Ahmed H. Mokhtar, Caleb Na, Maryam Mahjoob, David M.P. Di Fonzo, Jeffrey D. Mosko, Christopher W. Teshima, Gary R. May, Natalia Causada Calo

**Affiliations:** ^1^ Division of Gastroenterology St. Michael's Hospital, University of Toronto Toronto Canada; ^2^ Temerty Faculty of Medicine University of Toronto Toronto Canada; ^3^ Department of Internal Medicine McGill University Health Centre Montreal Canada

**Keywords:** ampullary lesions, ampullectomy, bleeding, endoscopic resection, endoscopic retrograde cholangiopancreatography

## Abstract

**Introduction and objectives:**

Endoscopic ampullectomy is the preferred treatment for selected periampullary lesions, yet up to 10.6% of patients may experience delayed bleeding post‐procedure. This study aims to identify predictors for bleeding, which remain poorly understood.

**Methods:**

This was a single‐center retrospective cohort study of adult patients who underwent endoscopic ampullectomy (EA) between January 2011 and September 2023. The primary outcome was the risk factors for delayed bleeding, defined as post‐procedural bleeding that necessitated either an emergency department visit, hospital admission, blood transfusion, or re‐intervention. Secondary outcomes included adverse events, such as perforation and pancreatitis.

**Results:**

A total of 113 patients underwent EA, and 25 (22.1%) experienced delayed bleeding. Of these, 20 (80%) required repeat endoscopy, six (24%) needed blood transfusions, and three (12%) were managed conservatively. Multivariable logistic regression analysis identified international normalized ratio ≥1.2 (odds ratio [OR] 3.32, 95% confidence interval [95% CI] 1.03–10.74, *p* = 0.05), presence of high‐grade dysplasia or intramucosal cancer (OR 3.76, 95% CI 1.20–11.81, *p* = 0.03), female sex (OR 3.14, 95% CI 1.11–8.93, *p* = 0.03), size of lesion (OR 1.04, 95% CI 1.01–1.08, *p* = 0.03) and procedure duration (OR 0.98, 95% CI 0.97–0.99, *p* = 0.04) as independent predictors of delayed bleeding.

**Conclusion:**

Several factors, including features of high‐grade dysplasia‐intramucosal cancer, international normalized ratio ≥1.2, female sex, lesion size, and procedure duration are associated with delayed post‐ampullectomy bleeding. These factors should be taken into consideration when strategizing the reduction of post‐ampullectomy bleeding.

## INTRODUCTION

Periampullary lesions represent 10% of all duodenal tumors.^1^ Historically, surgical resection was the primary treatment modality for periampullary lesions.^2^ However, it is associated with a 10% risk of mortality, and up to 65% risk of morbidity,^2^ including pancreatic fistulas, internal hemorrhage, and wound infection.^3^ The detection of periampullary lesions has increased due to more widespread upper gastrointestinal endoscopy and improved optical technology.^4^ Most of these lesions have a benign morphology or are present at an early stage, providing an opportunity for less‐invasive curative resection approaches.^5^, ^6^ Specifically, endoscopic ampullectomy (EA) has become the preferred resection modality in these selected patients.^7^ However, this is not without risk, as complications such as bleeding may occur both intra‐procedurally or manifest as delayed hemorrhage.^8^, ^9^

The intricacies of the ampullary region, are characterized by a rich vascular network and proximity to major vessels. Hypothetically, the bleeding after the procedure can come from both sources, namely, miniature transected branches of the gastroduodenal artery in this area, but also small capillary veins from the submucosal space, heightening the risk of hemorrhagic events.^4^, ^10^ Indeed, the risk of post‐ampullectomy bleeding has been reported in up to 10.6% of cases in different series. It requires vigilant postoperative monitoring and timely intervention^10^, ^11^, ^12^, ^13^ As such, the objective of this study was to identify and investigate the predictors for delayed bleeding in the endoscopic treatment of periampullary lesions.

## METHODS

### Study design

This was a retrospective cohort study of adult patients referred to a high‐volume tertiary care center for the endoscopic resection of a lesion of the major papilla, between January 2011 and December 2023. Cases were identified through a prospectively maintained endoscopic retrograde cholangiopancreatography registry. The registry was approved by the Institutional Research Ethics Board (REB #23‐142).

### Study population

Adult patients (> 18 years of age) requiring EA for the treatment of ampullary lesions were included. Lesions may have been detected through endoscopy and staged with various imaging modalities, including initial endoscopic assessment, abdominal ultrasound, CT scan, magnetic resonance cholangiopancreatography, and endoscopic ultrasound, particularly to determine intraductal extension which had to be < 1 cm. Characteristics of lesions that were considered favorable for benign morphology included features such as those confined to the mucosa, a soft consistency, lack of an ulcerative surface or spontaneous bleeding, lack of malignant features in advanced imaging, and lesions with less than 1 cm of intraductal growth.^14^, ^15^ All patients included in this study had superficial targeted biopsies taken as part of the evaluation process prior to EA. Among all patients, the biopsies revealed low‐grade dysplasia (LGD) in 83 patients, high‐grade dysplasia (HGD) in 28 patients, and Tis in two patients. Treatment for all patients, including those diagnosed as Tis, was proposed based on multidisciplinary consensus, and after patients’ agreement. No patients underwent EA for diagnostic purposes. Patients with incomplete data records were excluded. Data, including patient demographics, procedural indications, procedural success, and adverse events, were extracted from the registry.

### Objectives

The primary objective was to identify independent predictors of delayed post‐ampullectomy bleeding. The secondary outcomes were time‐to‐delayed bleeding, length of hospital stay, and other adverse events, including perforation and pancreatitis.

### Outcome definition

Delayed bleeding was defined as all bleeding events occurring after completing the procedure till the first month following EA confirmed endoscopically and requiring additional management including repeat endoscopy in cases of a visible active source of bleeding in the post‐ampullectomy area or visible transected vessels which could be a potential cause of bleeding in correspondence to clinical manifestation. Also, supportive treatment, transfusion, interventional radiology treatment, repeat hospitalization, or emergency department visits.^16^

### Procedure description

Procedures were performed by two expert endoscopists with over 15 years of experience in EA under general anesthesia or conscious sedation, depending on the patients’ procedural tolerance and lesion morphology. A duodenoscope was used for the entire procedure. The endoscopic assessment of the ampulla was performed in all cases to exclude malignant features, namely, deep invasion features based on lesion morphology and vascular and surface patterns. In all cases, the bile duct was cannulated for the assessment of intraductal extension. Where possible, the pancreatic duct was cannulated for evaluation of its anatomy and further guidance for pancreatic duct stent placement. In select cases, a submucosal injection consisting of methylene blue and saline was performed to the inferior aspect of the periampullary lesions, to facilitate visualization and ensure complete resection. Standard, medium‐stiffness, braided polypectomy snares (typically 15 mm, Olympus SnareMaster Plus), were utilized. A blended electrosurgical current was employed for all lesions (Erbe electrosurgical generators with the setting of Endocut Q, effect 3). When possible, the goal was to obtain an en‐bloc resection. For laterally spreading lesions, piecemeal resection was usually required. For lesions with < 1 cm intraductal involvement, biliary sphincterotomy and extraction balloon were used to expose the intraductal component from within the common bile duct. Snare resection was utilized to remove any intraductal growth if present. In cases of intraprocedural bleeding or visible transected vessels at the resection base, the management and prophylaxis of bleeding was made by epinephrine injection, hemostatic clips placement, or coagulation by the tip of the snare or argon plasma coagulation, at the discretion of endoscopists. Most cases had a pancreatic stent placed. Specimens were retrieved with a Roth net (US Endoscopy) and sent for histopathology analysis. After the procedure, all patients were admitted for observation with a similar post‐procedural management strategy involving overnight fasting, an intravenous infusion of proton pump inhibitor, followed by diet advancement the following day if the patient was well. Antibiotics were not routinely administered. Immediate bleeding was addressed with snare‐tip soft coagulation or coagulation graspers (Coagrasper; Olympus). An indomethacin suppository was routinely administered post‐procedure for pancreatitis prophylaxis. In cases of delayed bleeding, the step‐up treatment strategy was utilized, starting with a repeat endoscopy, followed by interventional radiology and surgical treatment if prior options failed. A blood transfusion was administered depending on the patient's clinical condition and was managed on a case‐to‐case basis.

### Statistical analysis

Descriptive statistics were reported as mean with standard deviations or percentages with 95% confidence intervals. Categorical variables were compared with chi‐square. To assess for factors associated with delayed bleeding, variables were divided into three broad categories: patient‐, lesion‐, and procedure‐related. Variables were chosen based on biological plausibility and previous literature and the intent of the logistic regression analysis was to identify predictors of delayed bleeding and was not conceptualized to infer causation of bleeding. The patient‐related variables were biological sex, age, and use of antithrombotic drugs; lesion‐related variables included: lesion morphology as per the Paris classification, lesion size, and histology. International normalized ratio (INR) was also included as a parameter of coagulation status. Importantly, most patients had INRs that were close to normality to safely undergo the procedure. The distribution of INR values was binomial in our sample and hence, the cutoff point of 1.2 was chosen to enter it in the logistic regression. Procedure‐related variables included: procedure duration, intraprocedural bleeding, type of resection (en bloc vs. piecemeal), and prophylaxis of bleeding (use of clips, cautery, injection) in the absence of intraprocedural bleeding. Variables were introduced, one at a time, into a multivariable logistic regression model. Logistic regression estimates were expressed as odds ratio (OR) with 95% confidence intervals. Receiver operating characteristic (ROC) analysis was performed to describe the accuracy of the prediction model (logistic regression). Cox Proportional Hazards Regression was used to examine the time‐to‐event data for the main outcome. Additionally, survival analyses were performed to construct Kaplan‐Meier curves and log‐rank tests to visualize and compare event‐time distributions. All statistical tests were two‐sided and considered significant at *p* < 0.05. Statistical analysis was performed with STATA v. 18.0.

## RESULTS

### Baseline characteristics

This study included 113 consecutive patients (54.9% female, mean age 66.2 ± 12.2 years) with confirmed adenomas with dysplasia (LGD = 83 and HGD = 28) or carcinoma in situ (two patients), who underwent EA. The mean lesion size was 27.0 ± 14.3 mm and most lesions were described as 0‐Is (96; 85.0%) as per the Paris classification.^17^ The common bile duct was involved in 12 cases (10.6%). Further clinical and lesion characteristics are presented in Tables 1 and 2.

**TABLE 1:** Patient's characteristics.

	All patients *n* = 113	Patients with delayed bleeding *n* = 25	Patients without delayed bleeding *n* = 88	*p*‐value
**Age (years, mean, SD)**	66.2 ± 12.2	65.8 ± 13.2	66.4 ± 12.0	0.82
**Women (*n*, %)**	51 (45.1)	15 (60.0)	36 (40.9)	0.09
**Comorbidities (*n*, %)**				
Hypertension	49 (43.3)	15 (60.0)	34 (38.6)	0.06
Diabetes mellitus	19 (16.8)	5 (20.0)	14 (15.9)	0.63
Dyslipidemia	35 (31.0)	11 (44.0)	24 (27.3)	0.11
Coronary artery disease	10 (8.8)	3 (12.0)	7 (8.0)	0.53
Obesity	5 (4.4)	1 (4.0)	4 (4.5)	0.91
Cirrhosis	2 (1.8)	1 (4.0)	1 (1.1)	0.34
Peripheral arterial disease	5 (4.4)	1 (4.0)	4 (4.5)	0.91
Arrhythmia	8 (7.1)	1 (4.0)	7 (8.0)	0.50
Thrombosis	5 (4.4)	0 (0)	5 (5.7)	0.22
Prior diagnosis of cancer	18 (16.1)	3 (12.0)	15 (17.1)	0.54
**Laboratory Tests**				
INR (median (IQR))	1.1 (1.1–1.2)	1.20 (1.1–1.3)	1.1 (1.0–1.1)	0.01
Platelet Count	213.8 ± 64.6	215.3 ± 61.3	208.3 ± 76.2	0.63
Creatinine	85.3 ± 28.9	81.8 ± 20.7	86.3 ± 30.1	0.49
Hemoglobin	123.1 ± 18.0	120.6 ± 21.2	123.8 ± 17.1	0.44
Bilirubin	14.9 ± 8.4	16.9 ± 11.9	14.3 ± 7.1	0.17
**ASA classification**				
ASA 1 & 2	69 (61.10)	20 (80.0)	49 (55.70)	0.03
ASA 3 & 4	44 (38.9)	5 (20.0)	39 (44.3)	
**Anti‐thrombotic medications** Anticoagulants antiplatelets	22 (19.9) 9 (40.9) 13 (59.1)	6 (24.0) 4 (66.7) 2 (33.3)	16 (18.2) 11 (68.75) 5 (31.25)	0.52

**TABLE 2:** Baseline lesion and procedure characteristics.

Baseline lesion and procedure characteristics	Total	Patients with delayed bleeding (*n* = 25)	Patients without delayed bleeding (*n* = 88)	*p*‐value
**Size (mm)** (median (IQR))	21.0 (15.0–30.0)	30.0 (20.0–40.0)	20.0 (15.0–30.0)	0.09
**Paris classification**				
IIa	17.0 (15.0)	4.0 (16.0)	13.0 (14.8)	0.02
Is	96.0 (85.0)	21.0 (84.0)	75.0 (85.2)	
**Genetic predisposition**				
Sporadic	105.0 (92.9)	24.0 (96.0)	81.0 (92.1)	0.46
FAP	8.0 (7.1)	1.0 (4.0)	7.0 (7.8)	
**Resection**				
En‐bloc	56.0 (49.6)	10.0 (40.0)	46.0 (52.3)	0.30
Piecemeal	57.0 (50.4)	15.0 (60.0)	42.0 (47.73)	
**Biliary Sphincterotomy performed**	33.0 (29.2)	10.0 (40.0)	23.0 (26.1)	0.18
**Ductal involvement**				
CBD involvement	12.0 (10.6)	4.0 (16.0)	8.0 (9.1)	0.32
CBD stent placed	71.0 (62.8)	15.0 (60.0)	56.0 (63.6)	0.74
PD stent placed	98.0 (86.7)	23.0 (84.0)	77.0 (87.5)	0.65
**Pathology**				
HGD/cancer	65.0 (57.2)	19.0 (76.0)	46.0 (52.3)	0.03
**Pancreatitis**	19.0 (16.8)	2.0 (8.0)	17.0 (19.3)	0.18
**Perforation**	7.0 (6.2)	2.0 (8.0)	5.0 (5.7)	0.67
**Length of hospital stay (days)** (median (IQR))	5.0 (4.0–6.0)	7 (6.0–8.0)	4.0 (3.0–5.3)	<0.01
**Procedure duration (min) (median (IQR))**	60 (42.0–70.0)	47 (40.0–60.0)	60 (45.0–75.0)	0.03

### Endoscopic ampullectomy

The resection approach as en‐bloc or piecemeal was determined by the morphology of the lesion, and both methods were comparably performed (49.6% and 50.4%, respectively). Biliary sphincterotomy was performed in 33 (29.2%) cases. CBD involvement was confirmed in 10.6% of patients. Biliary and pancreatic stent placement was performed in 62.8% and 86.7% of cases, respectively. Intraprocedural bleeding occurred in 79.6% of cases and the mean procedure duration was 60 (interquartile range [IQR]: 42–70) minutes. Details regarding the procedure are described in Table 2.

### Outcomes

There were 25 (22.1%) patients delayed bleeding (Table 2). Delayed bleeding occurred at a median of 24 h (IQR: 6–24) post‐procedure. Twenty‐one patients (84%) experienced bleeding within 24 h, while only four (16%) experienced delayed bleeding after 24 h. For the management of bleeding, 20 (80%) patients required repeat endoscopic intervention, six (24%) required blood transfusions and five (20%) were managed conservatively. Patients who experienced delayed bleeding had a longer hospital stay (7.0 [IQR: 6.0–8.0] days) than those without (4.0 [IQR: 3.0–5.3] days).

A multivariable logistic regression model revealed that the factors independently associated with delayed bleeding included: INR ≥1.2 (OR 3.32, 95% confidence interval [95% CI] 1.03–10.74, *p* = 0.05); HGD/cancer (OR 3.76, 95%CI 1.20–11.81, *p* = 0.03); female sex (OR 3.14, 95% CI 1.11–8.93, *p* = 0.03), lesion size (OR 1.04, 95% CI 1.01–1.08, *p* = 0.03); procedure duration (OR 0.98, 95% CI 0.97–0.99, *p* = 0.04; Table 3). This regression model predicts the risk of delayed bleeding accurately 76.5% of the time as shown in the ROC for the diagnostic performance of the model (Figure S1).

**TABLE 3:** Multivariable analysis for post‐ampullectomy delayed bleeding.

Characteristic	Multivariate OR for delayed bleed (95% CI)	Standard error	95% Confidence interval	*p*‐value
HGD or cancer	3.76	2.19	1.20–11.80	0.02
Female sex	3.15	1.67	1.11–8.93	0.03
INR ≥1.2	3.32	2.00	1.02–10.73	0.05
Size of lesion (mm)	1.04	0.02	1.01–1.10	0.03
Procedure duration (min)	0.98	0.01	0.97–0.99	0.04

**FIGURE 1:**
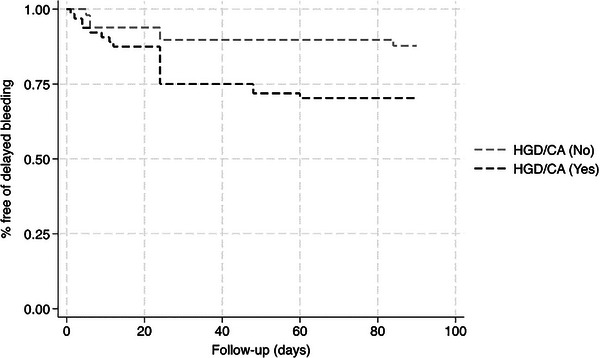
Time‐to‐delayed bleeding Kaplan‐Meier curve.

In addition, a multivariable Cox regression analysis was performed to determine time‐to‐event risks and to identify any factors that predict delayed bleeding with respect to time. The sole factor associated with time‐to‐bleeding was HGD/cancer (hazard ratio 2.6, 95% CI 1.03–6.83). Kaplan Meier curves were constructed (Figure 1). Overall, adverse events included perforation (*n* = 7, 6.3%) and pancreatitis (*n* = 19, 16.8%), which in most cases had a mild course. There were no deaths in this cohort at 30 days.

## DISCUSSION

Delayed bleeding is a well‐recognized adverse event after EA. Although in most cases the course is moderate in severity, management requires additional resources and increases hospital length of stay.^11^ In this retrospective cohort study, factors that predicted delayed bleeding included features of HGD or cancer, INR ≥1.2, female sex, size of the lesion, and procedure duration. Furthermore, patients who experienced delayed bleeding had a longer hospital stay and 80% required endoscopic re‐intervention to control the bleeding.

While there are numerous studies pertaining to the safety and efficacy of EA, there is a dearth of literature reporting on the risk factors for delayed bleeding. This study revealed that HGD or early cancer as compared with LGD might potentially enhance the occurrence of delayed bleeding. In addition, this was the sole factor that was independently associated with a faster time‐to‐bleeding with an adjusted hazard ratio of 2.6 (95% CI 1.03–6.83). Meunier al.,^18^ identified a trend toward an increased risk of adverse events in the case of malignant histology. Similarly, Jiang et al.,^19^ in their study showed that patients with high‐grade intraepithelial neoplasia (HIN) in removed ampullary lesions, were more likely to have post‐ampullectomy complications in general (OR 6.52, 95% CI 1.45–46.77, *p* = 0.027). This finding may be related to a hypothetically increased neovascularization in more advanced lesions. However, it is important to note that this association is based on post‐procedural pathology, which limits its utility for predicting bleeding risk prior to the procedure unless the histology can be confidently determined a priori. Ideally, pre‐procedural thorough evaluation with digital chromoendoscopy could provide critical insight into the lesion's histology.^20^ Moreover, the endoscopic surface and pit pattern of ampullary adenomas differs from colorectal adenomas and the correlation with the final pathology in the specimen analysis is less reliable. Given that, further efforts are needed to help improve pre‐procedural ampullary adenoma assessment and histological prediction.

Another relevant predictor of delayed bleeding was INR ≥1.2. The level of 1.2 as a cut‐off was equivocal only to the upper normal limit of INR as most patients had their INR corrected prior to resection. Despite this, a slight increase in the INR may be associated with a greater risk of delayed bleeding. In 84% of patients, bleeding occurred within 24 h of EA, highlighting the importance of close monitoring in this early period. The risk of delayed bleeding significantly diminished after 24 h, particularly those with an INR <1.2, indicating that these patients may be able to be discharged with a hospital length of stay of 1 day. In terms of potential causes for INR ≥1.2, the use of antiplatelet or anticoagulant medications was not associated with delayed bleeding. In our study, only six (24%) patients among whom delayed bleeding occurred, were on pre‐procedural anticoagulation (two on dual antiplatelet therapy and four on low doses of aspirin of 81 mg). In addition, in all cases medications were held according to local standards, namely, our practice is to withhold anticoagulation medications for 3–5 days prior to an EA, depending on the medication.^21^, ^22^ Although clinical practice often involves discontinuing anticoagulants when a patient is at increased risk of bleeding, our study found that even within the recommended INR range, slight elevations can still pose problems. Specifically, an INR ≥1.2 was identified as a predictor of delayed bleeding, despite most patients having their INR corrected before the procedure. This suggests that while stopping anticoagulants is crucial, managing INR levels within a safe range remains critical to minimizing bleeding risks. Conversely, Meunier et al.,^18^ demonstrated that anticoagulation was independently related to delayed bleeding after an EA (OR 4.37, 95% CI 2.86–5.95). That discrepancy might be related to the fact that our sample size was relatively small, and medications were stopped prior to the procedure. This discrepancy suggests that elevated INR could be influenced by other factors, such as underlying coagulopathy or procedural trauma, rather than solely by anticoagulant therapy. Nonetheless, the mild elevation in the INR as a predictor of bleeding still points out that coagulation dysregulations (either medically or pharmacologically) are likely important determinants in the risks of bleeding. Therefore, as ampullectomy remains a high‐risk procedure with a risk of delayed bleeding, following guidelines for peri‐procedural management of anticoagulation is of utmost importance.^23^

Lesion size (particularly exceeding 20 mm) was another relevant predictor for the occurrence of delayed bleeding. A previous study reported a 7% increase in the odds of bleeding with each additional cm in size of an ampullary adenoma (OR 1.07 95% CI 1.01–1.13).^18^ Larger lesions require higher vascular supply, regardless of the underlying histology. The resection of larger lesions might theoretically cause larger submucosal damage and further vessel injury, which might cause delayed bleeding. This is also observed after large colonic polypectomy, as described by Burgess et al.^24^

Interestingly, prolonged procedure time was associated with a lower odds of bleeding. This may indicate that bleeding vessels could potentially be treated intraprocedurally in longer resections and prevent delayed bleeding, particularly if the injectate contained epinephrine. The effect of the epinephrine may reduce intraprocedural bleeding temporarily and bleeding may become evident once this effect dissipates; depending on the procedure duration, the bleeding may be missed.

Finally, our study did not find any significant associations between delayed bleeding and other variables, such as age, or other procedural aspects such as en‐bloc resection, biliary sphincterotomy, stent placement, intraprocedural bleeding, or performed prevention of bleeding like coagulation or defect clipping. There are other studies that reported these variables as risk factors for delayed bleeding.^12^, ^19^, ^25^, ^26^, ^27^, ^28^ Moreover, technical factors such as the type of polypectomy snares and cautery settings were not routinely reported in our study and hence, they could not be analyzed, but could potentially impact bleeding risk. Previous data suggests that alternative techniques, such as using non‐braided snares and pure‐cut cautery, could mitigate bleeding risks.^29^, ^30^, ^31^ This further suggests that analysis of larger cohorts is important to ascertain patient‐specific and procedural factors truly influencing the risk of delayed bleeding to establish preventive strategies that optimize post‐procedural care.^32^, ^33^

Our study is not without limitations. First, the observational and retrospective nature of the study introduces inherent biases and confounding by indication, and the potential for incomplete data capture. Second, the single‐center design may limit generalizability to broader patient populations and centers. However, EA is a high‐risk procedure that should be performed in expert hands and in tertiary centers. Third, the relatively small sample size might affect the study's power and limit the detection of other predictors. Lastly, the absence of standardized protocols for INR correction may introduce variability in preoperative management. Further studies with larger cohorts are required to validate our findings.

## CONFLICT OF INTEREST STATEMENT

1. All support for the present manuscript (Funding, provision of study materials, medical writing, article processing charges, etc.) – None

2. Grants or contracts from any entity – None

3. Royalties or licenses – None

4. Consulting fees – Boston Scientific (CWT) and Olympus (GRM)

5. Payment or honoraria for lectures, presentations, speakers bureaus, manuscript writing, or educational events – Boston Scientific (JDM & CWT), Pendopharm (JDM), Vantage (JDM), Medtronic (CWT, JDM & GRM), Pentax (GRM) and Fuji (GRM)

6. Payment for expert testimony – None

7. Support for attending meetings and or travel – None

8. Patents planned, issued, or pending – None

9. Participation on the Data Safety Monitoring Board or Advisory Board – Pendopharm, Janssen, Pentax, Fuji, and Boston Scientific (JDM)

10. Leadership or fiduciary role in another board, society, committee, or advocacy group paid or unpaid – None

11. Stock or stock options – None

12. Receipt of equipment, materials, drugs, medical writing, gifts or other services – None

13. Other financial or non‐financial interests – None

## ETHICS STATEMENT

‐Approval of the research protocol by an Institutional Reviewer Board: Yes, (REB #23‐142).

## PATIENT CONSENT STATEMENT

N/A.

## CLINICAL TRIAL REGISTRATION

N/A.

## Supporting information




**Figure S1** Receiver operating characteristic (ROC) model.
