# Supplementary figures and images for: Factors associated with delayed bleeding following ampullectomy: A retrospective cohort study
Source: DEN Open. 2025 Feb 14;5(1):e70078. doi: 10.1002/deo2.70078 (PMC11827579; doi:10.1002/deo2.70078)

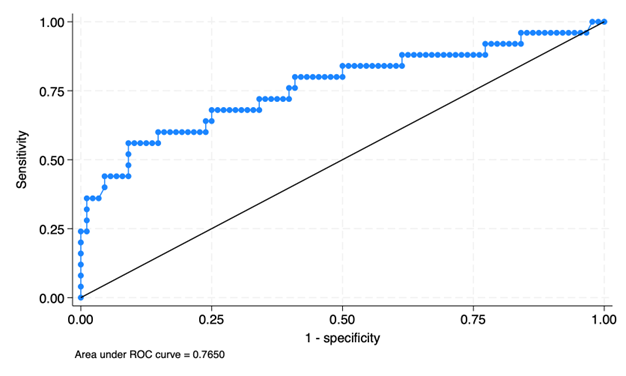

Supplement: Supplementary file 1 — Figure S1 Receiver operating characteristic (ROC) model. [file DEO2-5-e70078-s001.tif]
